# Overview and Diversity of Fungi of the Genus *Aspergillus* Section *Nigri* on Maize and Small Grains

**DOI:** 10.3390/foods14122146

**Published:** 2025-06-19

**Authors:** Milica Lučev, Jelena Stepanović, Vesna Kandić Raftery, Zoran Čamdzija, Ana Obradović, Milan Stevanović, Slavica Stanković

**Affiliations:** 1 Maize Research Institute Zemun Polje, Slobodana Bajića 1, 11185 Belgrade, Serbia; vkandic@mrizp.rs (V.K.R.); zcamdzija@mrizp.rs (Z.Č.); aobradovic@mrizp.rs (A.O.); mstevanovic@mrizp.rs (M.S.); sstojkov@mrizp.rs (S.S.); 2 Institute of Pesticides and Environmental Protection, Banatska 31b, 11080 Belgrade, Serbia; jelena.stepanovic@pesting.org.rs

**Keywords:** *Aspergillus welwitschiae*, *Aspergillus tubingensis*, calmodulin, RPB2, wheat, triticale, spelt

## Abstract

The presence of filamentous fungi with toxigenic ability from the *Aspergillus* genera is frequently found in maize kernels, and this can lead to decay and mycotoxin contamination of the kernels. In this study, we morphologically and molecularly characterized 45 isolates of *Aspergillus* section *Nigri* originating from maize and small grains (wheat, triticale, and spelt) in Serbia. Based on morphological traits, they were classified into two morpho groups. Representative isolates from both morpho groups were further molecularly characterized through sequencing of ITS, CaM and RPB2 genes in order to compare species composition, which could affect specific mycotoxicological risks. Morpho GroupI was molecularly identified as *Aspergillus welwitschiae* and morpho GroupII as *Aspergillus tubingensis*. Phylogenetic analysis of the CaM gene revealed that the Serbian *Aspergillus welwitschiae* isolate belongs to the H8 haplotype, while *A. tubingensis* isolates clustered into two subclusters. This is the first report of *A. tubingensis* as the causal agent of black mold of small grains (wheat, triticale and spelt) in Serbia. This distribution underscores the ecological preferences of species within the genus *Aspergillus* Section *Nigri* across various agricultural products. It emphasizes the importance of comprehending their occurrence, distribution, aggressiveness and potential for mycotoxin production in food safety assessments.

## 1. Introduction

The group known as Black Aspergilli, scientifically classified as *Aspergillus* section *Nigri*, comprises 27 species found across the globe. These fungi typically form dark colonies and produce conidial heads that are either uniseriate or biseriate [1]. *Aspergillus* section *Nigri* are fungi known for causing food spoilage and are found extensively in nature. While primarily sourced from soil, certain members of this group have been observed in various environments such as decaying organic material and on the surfaces of plants [2], contributing to food spoilage and diseases in maize [3], onions [4], grapes [5], and peanuts [6]. Strains of *Aspergillus* section *Nigri* can colonize maize and small grains (which include wheat, triticale, spelt, etc.) during pre-harvest, harvest, or post-harvest storage phases. Their ability to colonize diverse substrates underscores the importance of understanding their taxonomy, ecology, and potential impacts on agricultural and food safety.

*Aspergillus* section *Nigri* poses significant challenges in species classification due to the highly similar morphology and chemical composition/traits between the species. Therefore, the inclusion of molecular methodology is indispensable for accurate identification [1,7]. The taxonomy of this group is in constant flux, with new species continually being described.

In the most recent update to the genus *Aspergillus*, section *Nigri* includes both biseriate and uniseriate species and is divided into five series: *Carbonarii*, *Nigri* (formerly the *A. niger* aggregate), *Heteromorphi*, *Japonici*, and *Homomorphi* [8]. Species commonly found in maize and small grains are part of the biseriate *Nigri* series, including *A. niger*, *A. welvitschiae* and *A. tubingensis*. A recent genomic analysis revisiting species boundaries within the *Nigri* series suggested a substantial diminution in the number of species, proposing that *A. niger* and *A. welvitschiae* may actually be the same species [9]. However, this suggestion has not been widely accepted, and a phylogenomic study by Steenwyk et al. [10] supported the existence of two distinct clades.

The magnitude of *Aspergillus* section *Nigri* species, particularly its appearance and potential for mycotoxin production, has been extensively studied globally [11,12,13,14,15]. The significance of these fungi as pathogens has been on the rise in the last decade, largely due to climate change. This trend not only heightens plant vulnerability but also facilitates the spread of these highly strident species [16,17].

Two of the mycotoxins vastly present in contaminated human food and animal feed, ochratoxin A (OTA) and fumonisin B2 (FB2), are usually associated with the *A. niger* aggregate. Nevertheless, worldwide studies have shown that only strains of *A. niger* and *A. welwitschiae* are capable of producing these two mycotoxins in both substrates, culture media and natural substrates [7,18]. OTA is known for its nephrotoxic and carcinogenic properties [19], while FB2 has demonstrated cytotoxic activities in animals [20].

Considering the importance of correct identification of pathogens, the primary objective of this study was to identify the causative agents responsible for the observed black mold on maize cobs, kernels and small grains, from which isolates were collected annually during the harvest period from 2013 to 2022 in Serbia. The second objective was to test for the diversity of these pathogens, which can contribute to the disease’s etiology. Therefore, a comprehensive investigation was conducted—the obtained isolates were identified and characterized through detailed multilocus analyses and morphological assessments.

## 2. Materials and Methods

### 2.1. Fungal Isolates

To examine the diversity of *Aspergillus* section *Nigri* species, isolates from the collection of the Laboratory of Phytopathology at the Maize Institute “Zemun Polje” were used. A total of 45 isolates were selected and collected over a ten-year period, from 2013 to 2022. These isolates originated from maize (30), wheat (5), triticale (5), and spelt (5) and were collected from eight locations across Serbia (Table S1). The isolates had been previously identified to the section level using conventional morphological methods, based on both macroscopic and microscopic characteristics. For further analysis, monosporial cultures were stored on PDA and MEA slants at 4 °C.

### 2.2. Morphological Identification

**Figure 1:**
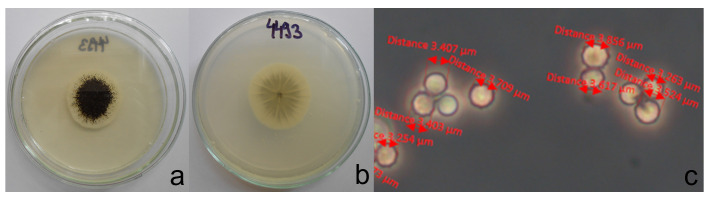
Colony morphology on PDA medium (representative of morpho group I-isolate 4493): (**a**)—obverse, (**b**)—reverse and (**c**)—morphology of conidia on MEA medium.

**Figure 2:**
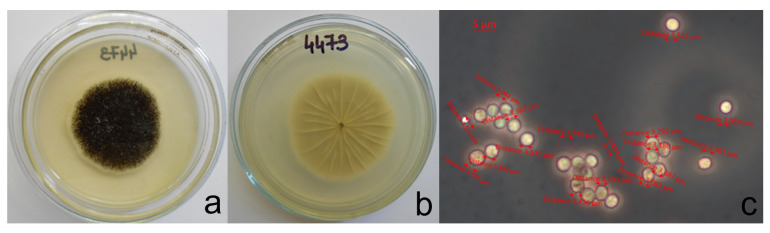
Colony morphology on PDA medium (representative of morpho group II-isolate 4473): (**a**)—obverse, (**b**)—reverse and (**c**)—morphology of conidia on MEA medium.

Mycelial plugs measuring 5 mm in diameter were taken from the edges of 7-day-old cultures grown on PDA and placed at the center of new PDA plates. The morphological analyses followed the guidelines outlined by [21]. For macro morphological observations (colony morphology and growth rates), isolates were inoculated on PDA (Figure 1 and Figure 2). Each experiment was conducted in triplicate. The plates were then incubated for 7 days at 25 °C in the dark. For micromorphological observations using optical microscopy, microscopic mounts were prepared from colonies grown on malt extract agar (MEA) for 7 days at 25 °C. The microstructures, including vesicles and conidia, were measured (Table S1).

### 2.3. Molecular Identification of Isolates and Phylogenetic Analyses

Representatives of two perceived morphogroups (one from morpho GroupI and nine from morpho GroupII) were further molecularly identified and characterized. Extraction of genomic DNA was conducted from 7-day-old fungal colonies grown on PDA or MEA medium at 25 °C with the QIAGEN DNeasy Plant Mini Kit (following manufacturer’s instructions) and modified 3% CTAB protocol [22].

Molecular identification of obtained isolates was performed through amplification of three genomic regions proposed for identification of *Aspergillus* species [21]—Internal Transcribed Spacer (ITS), Calmodulin (CaM) and RNA polymerase II second largest subunit (RPB2) with primer pairs ITS1/ITS4 [23], CMD5/CMD6 [24] and 5F/7CR [25], respectively employed. PCR conditions applied for amplification of each region were as described in [21], with modifications that conditions applied for RPB2 were the same as for CaM amplification in [21] and that all PCR reactions were performed on 40 cycles. All PCR reactions were performed in a final volume of 25 μL containing 1XDreamTaq PCR Master Mix (Thermo Scientific, Vilnius, Lithuania), 0.4 μM of each primer and approximately 50 ng of template DNA. Reactions performed without template DNA were considered as negative controls. All obtained amplicons were sequenced through a commercial service (Macrogen, Amsterdam, The Netherlands) in both directions with the same primers employed for their amplification. Obtained sequences are deposited in NCBI GenBank under accession numbers shown in Table 1.

Obtained sequences were assembled with Staden Package program Pregap4 [26] and aligned with Muscle incorporated in MEGA11 [27]. Subsequent to the manual inspection of sequences and alignment, BLAST analysis was performed using the NCBI’s BLAST algorithm (https://blast.ncbi.nlm.nih.gov/Blast.cgi, accessed on 28 Januar2025.) for comparison of Serbian strains and all publicly available sequences.

**Figure 3:**
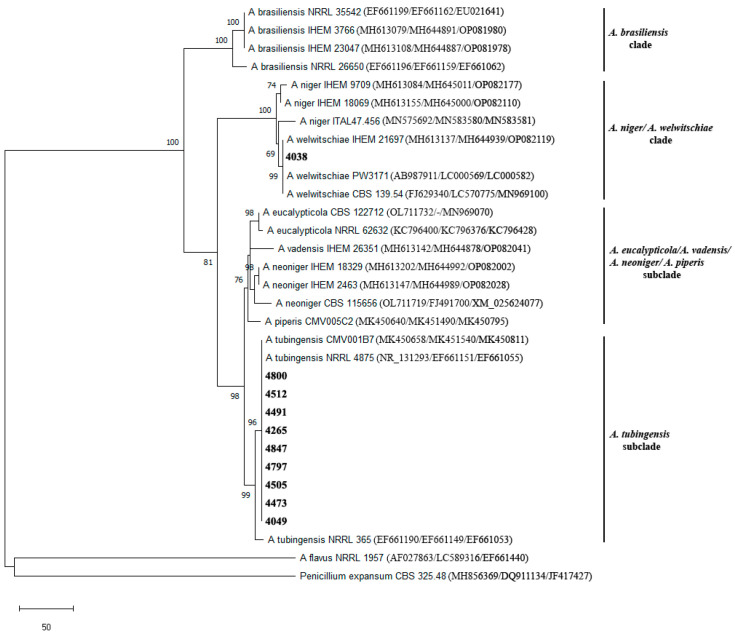
Maximum parsimony phylogenetic tree constructed of concatenated ITS, CaM and RPB2 sequences of representative isolates from section Nigri and Serbian isolates (only bootstrap values above 60% are presented on the tree). Serbian isolates tested in this study are marked in bold. GenBank accession numbers for ITS, CaM and RPB2 sequences are given in parentheses.

Additionally, in MEGA 11, a Maximum Parsimony (MP) phylogenetic species tree of concatenated ITS, CaM and RPB2 sequences was constructed using the Subtree-Pruning-Regrafting (SPR) algorithm with search level 1, 10 random replicates, 95% site coverage and a bootstrap test using 1000 replicates. In this analysis, 32 sequences were included—20 representative strains belonging to *Aspergillus brasiliensis*, *A. niger*, *A. welwitschiae*, *A. eucalypticola*, *A. vadensis*, *A. neoniger*, *A. piperis* and *A. tubingensis* species (all belonging to section *Nigri*) downloaded from NCBI’s GenBank, 10 isolates from this work and *A. flavus* and *Penicillium expansum* implemented as outgroup cluster (accession numbers of all sequences included in the analysis are given in Figure 3 and Table 1).

**Figure 4:**
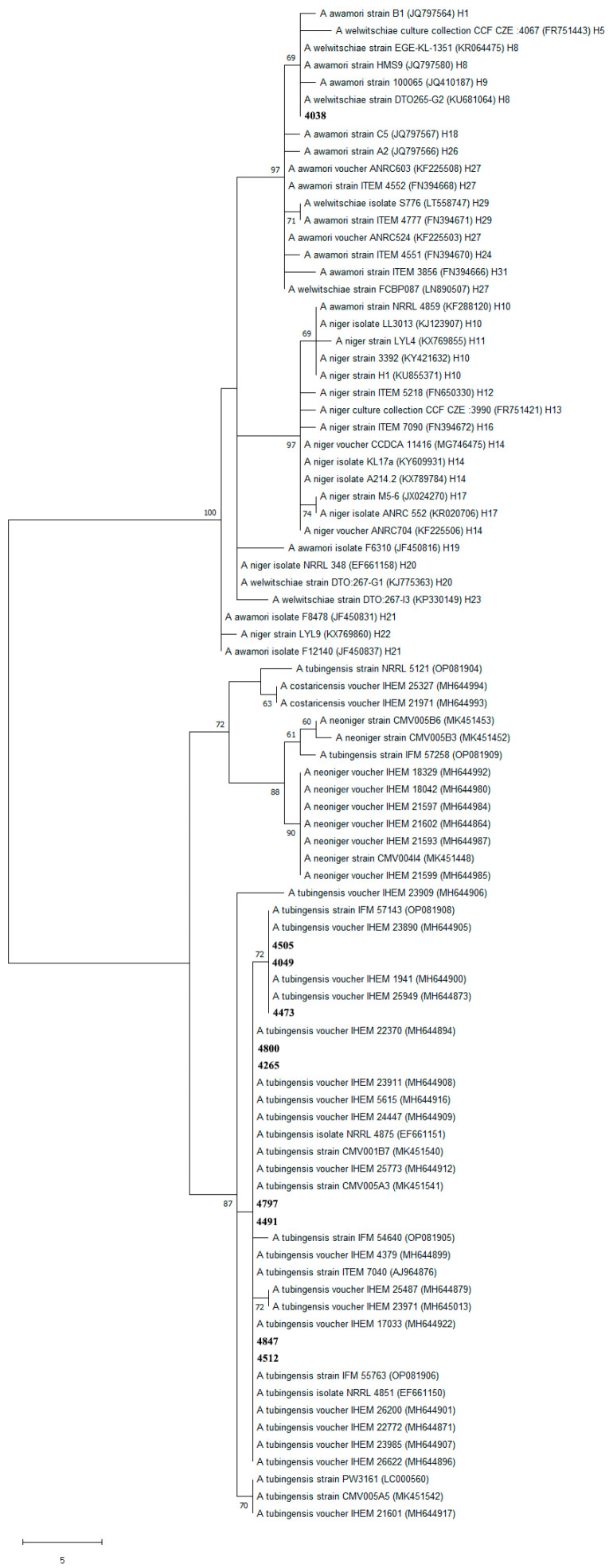
Maximum parsimony phylogenetic tree constructed of CaM sequences of representative isolates from section *Nigri* and Serbian isolates (only bootstrap values above 60% are presented on the tree). Serbian isolates tested in this study are marked in bold. GenBank accession numbers are given in parentheses; after accession number, haplotype affiliation is given if it exists [29].

In order to test to which CaM haplotype isolates from Serbia belong and are closest, the DnaSP 6.12.03 program [28] was used for haplotype identification, and an unrooted MP phylogenetic tree was constructed using MEGA 11 with the same conditions applied as in previous analysis, containing 88 sequences of publicly available strains belonging to *A. niger*, *A. welwitschiae*, *A. tubingensis*, *A. neoniger* and *A. costaricensis* species together with Serbian isolates (accession numbers of all sequences included in the analysis are given in Figure 4 and Table 1).

### 2.4. Pathogenicity Test

Artificial inoculation was performed with the method developed by Reid’s group [30]. Healthy maize hybrids were utilized to assess pathogenicity in field trials during the growing season (2022) and to satisfy Koch’s postulates, confirming the causal relationship between the organism and the disease. A selected group of 20 hybrids underwent inoculation by injecting a suspension of fungal spores into the silk channel. The spore suspension was prepared by flooding 7-day-old mycelium with 10 mL of sterile distilled water containing 0.01% Tween 20. The resulting suspension was then filtered through four layers of gauze under aseptic conditions to eliminate mycelial fragments. The concentration of the conidial suspension was standardized to approximately 10^7^ spores mL^−1^ using a hemocytometer, Neubauer, Bayern, Germany. Inoculation occurred three days after 50% of the plants reached the silking stage. In total, 2 mL of inoculum was injected through the silk channel into the upper ear of each plant. Control ears were inoculated with an equal volume of sterile distilled water. Each isolate was tested on five ears across four replicates, totaling 20 ears per isolate.

Visual assessments were conducted on a scale of 1 to 7 (1—complete absence of symptoms; 7—100% infected kernels), as previously described [30]. Subsequently, the pathogen was reisolated from the wounds developed on the inoculated ears to confirm its presence.

The pathogenicity test was conducted on wheat, triticale and spelt spikes under field conditions [31]. Cultures of the isolates, grown for seven days on potato dextrose agar (PDA) at 25 °C, were supplemented with 10 mL of sterile distilled water containing Tween 20 (0.01%). The inoculum was prepared by harvesting mycelia with a sterile scalpel, resulting in a conidial suspension. This suspension was filtered through a four-layer sterile gauze. Spore concentration was quantified using a hemocytometer and adjusted to 1 × 10^7^ spores/mL.

At the flowering stage, when more than 50% of the plants were in the full bloom phase, the spikes were artificially inoculated with a spore suspension of five isolates each, each at a concentration of approximately 1 × 10^7^ conidia/mL. In each repetition, groups of 20 spikelets were inoculated. The quantity of prepared inoculum was 20 mL per repetition. The control plants were treated with sterile water. The inoculum was applied with a hand sprayer on the surface of the flowering spikes. After inoculation ears are covered with wet PVC bags to create favorable moisture conditions for the development of fungal species. The bags were removed after 48 h.

Aggressiveness of the isolates (based on the number of infected spikelets per head) on wheat, spelt and triticale was determined after three weeks according to the scale of [32]. The scale represented the percentage of the spike surface showing visible disease symptoms: 1 = 0–5%, 2 = 5–15%, 3 = 15–30%, 4 = 30–50%, 5 = 50–75%, 6 = 75–90%, and 7 = 90–100%. Each number on the scale corresponds to the percentage interval of the area of classes with visible symptoms of the disease.

To test if there is statistical significance in virulence between the isolates, one-way ANOVA was performed using MSTAT on all isolates as well as on isolates from each host separately. Further on, to determine if there is a significant difference between mean virulence values of the isolates, Duncan’s multiple range test (DMRT) was applied in the same program.

The fungi were reisolated from symptomatic spikelets and were morphologically identical to the original isolates, thus fulfilling Koch’s postulates.

## 3. Results

### 3.1. Morphological Identification

Based on their colony appearance and morphological characteristics, we provisionally identified 45 isolates as members of the black *Aspergillus* and segregated them into Group I (5 isolates) and Group II (40 isolates). Group I was morphologically identified as *A. niger* based on its black colony color, biseriate conidial heads, and small conidia (2.9–3.9 μm), vesicle size (48–78 μm) characteristics consistent with descriptions by [33,34,35,36] (Table S1). Group I isolates typically formed compact black colonies with a greyish central region, occasionally showing slight yellowish tones and a brighter reverse colony appearance (Figure 1). In contrast, Group II isolates exhibited uniformly black to greyish-black colonies with maximal growth on plates (Figure 2). The diameter of conidia in isolates belonging to group II was in the range 3–3.8 μm. The vesicle size was 42–76 μm (Table S1).

Although there were slight differences in colony appearance between Group I and Group II, Group II typically produced uniformly black to greyish-black colonies.

### 3.2. Molecular Identification and Phylogenetic Analysis

Based on morphological characteristics, 45 fungal strains were initially identified as *Aspergilli* belonging to sect. *Nigri* and grouped into two groups. One isolate 4038 from Group I (which encompassed five isolates) and nine isolates (4049, 4265, 4473, 4491, 4505, 4512, 4797, 4800 and 4847) from Group II (which encompassed 40 isolates) were chosen for further molecular identification through ITS, RPB2 and CaM gene sequencing. BLAST analysis of ITS sequences confirmed that all ten Serbian isolates belong to *Aspergillus* species from section *Nigri* while CaM and RPB2 sequences identified isolate 4038 as *A. welwitschiae* (it shares 100% identity with *A. welwitschiae* voucher strain IHEM 22379-MH644968 in CaM and 99.9% with *A. welwitschiae* culture collection strain CBS:139.54-MN969100 in RPB2) and the other nine strains as *A. tubingensis* (they share 99.82–100% identity with *A. tubingensis* voucher strain IHEM 6184-MH644895 in CaM and 99.4–100% identity with *A. tubingensis* isolate NRRL 62643-KC796436 in RPB2).

MP phylogenetic analysis of concatenated ITS, CaM and RPB2 sequences of representative isolates from section *Nigri* and Serbian isolates in the final dataset included 2458 positions and resulted in six equally parsimonious trees obtained. Isolate 4038 clustered with a high bootstrap value of 99 together with representative *A. welwitschiae* isolates in a separate subclade inside *A. welwitschiae/A. niger clade* (with bootstrap value of 100) while the other nine tested Serbian strains clustered in a separate subclade also with a bootstrap value of 99 with representative strains of *A. tubingensis* inside *A. eucalypticola*/*A. vadensis*/*A. neoniger*/*A. piperis*/*A. tubingensis* clade (this clade showed a bootstrap value of 98) (Figure 3). This phylogenetic analysis confirmed the identification of isolate 4038 from morphological Group I as *A. welwitschiae* and isolates 4049, 4265, 4473, 4491, 4505, 4512, 4797, 4800 and 4847 from morphological Group II as *A. tubingensis*.

The analysis of CaM haplotype of Serbian isolates and publicly available strains belonging to *A. niger*, *A. welwitschiae*, *A. tubingensis*, *A. neoniger* and *A. costaricensis* species consisted of 34 haplotypes and in the final dataset of the constructed MP phylogenetic tree included 454 positions. Analysis resulted in the reconstruction of ten equally parsimonious trees in which isolate 4038 clustered together with *A. welwitschiae* haplotypes H1, H5, H8 (to which it belongs) and H9 with a not-so-high bootstrap value of 69. Some of the isolates that belong to haplotype H8 are from *Vitis vinifera* from Iran (KU681064), soybeans from Argentina (KF225510), dried figs from Turkey (KR064475), etc. Isolates 4049, 4473 and 4505 clustered together with *A. tubingensis* isolates from human ear and sputum as well as with isolate from dust from mattress (MH644905, MH644873 and MH644900) with bootstrap value of 72, while isolates 4265, 4797, 4491, 4847, 4512 and 4800 clustered together with a culture type strain of *A. tubingensis* NRRL 4875 (EF661151), isolate from walnut kernels of *Juglans regia* from South Africa (MK451540), isolate from a vineyard from Yamanashi in Japan (OP081906), etc. (Figure 4)

### 3.3. Pathogenicity Test

The pathogenicity test was performed in Zemun Polje on the same maize hybrid from which the fungal species were isolated. Pathogenicity of the isolates was confirmed through artificial inoculation of maize ears. Disease symptoms appeared on all inoculated ears and were identical to those observed in ears collected from various locations. The colony appearance and morphological characteristics of isolates recovered from inoculated ears matched those of the original isolates, confirming their pathogenicity and fulfilling Koch’s postulates, which establish a causal relationship between an organism and a disease. The control ears were symptomless. Under artificial inoculation conditions in the study year (2022), the tested isolates exhibited disease intensity levels ranging from 1.90 to 3.25. Isolate 4038, identified as *A. welwitschiae*, exhibited the lowest virulence on the maize ears (1.90), while the highest virulence (3.25) was observed in isolate 4491, identified as *A. tubingensis*.

Following artificial inoculation of wheat, triticale and spelt spikes, the first symptoms appeared during the grain formation and ripening stages. Infection at the flowering stage spread to the spikes, leading to premature ripening of the grains. The pathogen was successfully reisolated from the inoculated wheat spikes. The colony appearance and morphological characteristics of the isolate matched those of the original pathogen, thereby confirming Koch’s postulates. No symptoms were observed on the control spikes inoculated with sterile distilled water. All isolates tested showed pathogenicity on wheat, triticale and spelt.

One-way ANOVA showed that there are significant differences between the virulence of isolates on four plant species, i.e., maize, wheat, triticale and spelt. When analyzed separately, similar results were obtained on each species (Table S2).

Duncan’s multiple range test (DMRT) was applied to determine significant differences between mean virulence values of the isolates. The mean values of the isolates differed significantly, both when all species were considered together and when analyzed separately by species. The mean virulence values were generally highest for maize (1–30 isolates), while the lowest were for isolates obtained from spelt (41–45). The overall mean value for maize was 2.567, wheat 2.453, triticale 2.26 and spelt 1.973 (Table S3).

## 4. Discussion

Previous studies on black *Aspergillus* species from various substrates and hosts have highlighted the morphological similarities between *A. niger* and *A. tubingensis* [37,38]. These species share several morphological similarities, particularly in their colony appearance and conidiogenesis, making them difficult to differentiate based solely on macroscopic features. Both species typically form dark, black colonies on various culture media, although the colony size and texture can vary depending on the growth conditions. However, differentiation between *A. niger* and *A. tubingensis* is sometimes based on the production of sclerotia, a feature that was not observed in this study. According to Samson et al. [39], the formation of sclerotia is not a universal trait among *A. tubingensis* isolates and is observed only in certain strains, making it an unreliable criterion for distinguishing between the species. Microscopically, they exhibit similar conidiophore structures, with smooth black conidia arranged in a characteristic vesicular pattern.

In 2009, Martinez-Culebras et al. [40] and Bisbal et al. [41] studies showed that *A. niger* isolates can be successfully differentiated from *A. tubingensis* on the basis of ITS sequences while *A. welwitschiae* (former *A. awamori*) clustered together with *A. niger* and therefore could not be differentiated from it. Later in 2014, Samson et al. [21] in their profound study of the genus *Aspergillus* phylogeny suggested calmodulin as the second molecular marker (after ITS) because of its universal primers, the NCBI’s dataset containing sequences of almost all species and large accessibility for amplification. On the basis of CaM sequences, *A. welwitschiae* can be differentiated from *A. niger*. Their recommendation for the third marker was either β-tubulin (which can easily amplify paralogous genes) or RPB2, which is slightly difficult for amplification. Therefore, in our study ITS, CaM and RPB2 genes were administered in order to successfully identify our isolates from maize and small grains.

Our molecular identification revealed that *A. tubingensis* was the predominant species of *Aspergillus* section *Nigri* on maize, wheat, triticale and spelt in Serbia over the studied period from 2013 to 2022, which is in agreement with research conducted in other countries.

The variation in the distribution of black aspergilli species across Europe appears to be influenced by meteorological conditions and geographical factors. *A. tubingensis* and *A. niger* were found to be the predominant species across all countries studied. In contrast, *A. carbonarius* seems to be more prevalent in southern Mediterranean regions such as southern France, southern Italy, Portugal, and Greece [42]. In the Midwestern United States, while identifying black aspergilli isolates from maize, they identified the presence of *A. tubingensis* among the species [43]. Researchers from Italy identified 12 of the 30 isolated strains as *A. welwitschiae*, and 18 as *A. tubingensis*. In this limited sample, *A. tubingensis* and *A. welwitschiae* emerged as the predominant species isolated from maize kernels. However, a more comprehensive analysis is necessary to fully characterize the diversity of black Aspergilli found on maize kernels and to assess their potential contribution to fumonisin (FB) contamination [44]. This underscores the need for broader studies to better understand the distribution and impact of these fungal species on maize and food safety.

In Spain, ref. [45] identified species of the genus *Aspergillus* section *Nigri* on different matrices were studied. Grapes were identified as the primary source of *A. niger* aggregate isolates. Specifically, *A. tubingensis* was the most frequently isolated species from Spanish grapes, with 67% of isolates, followed by *A. welwitschiae* with 27% and *A. niger* with 5%. *A. welwitschiae* was the most abundant in grapes, accounting for 34% of the isolates, while *A. niger* was more often associated with maize and corn borer (46.6%) compared to grapes (10%). *A. tubingensis* showed consistent occurrence in grapes (24%) and cereals, particularly wheat (25.1%).

Cereals also serve as significant sources of *A. niger* aggregate isolates globally. Notably, *A. tubingensis* was highly prevalent in cereals, constituting 80%, 92%, and 91% of isolates from maize, wheat, and barley, respectively. The analysis conducted in Spain highlighted *A. tubingensis* as the most prevalent species across various substrates, notably cereals and grapes, although it was also found in other substrates. In contrast, *A. niger* and *A. welwitschiae* were present in these substrates but at lower frequencies compared to *A. tubingensis* [45].

In a study conducted in two countries, Italy and the USA, a precise assessment of the species composition of black Aspergilli strains isolated from collected maize samples using a DNA-based approach was presented [46,47]. Molecular identification revealed that *A. niger*, *A. welwitschiae* and *A. tubingensis* were the predominant species of *Aspergillus* section *Nigri* on maize in both countries, although their relative frequencies differed. In Italy, about 60% of the strains were *A. niger*/*A. welwitschiae* and 40% of *A. tubingensis*, while in the USA the composition was approximately 73% *A. niger*/*A. welwitschiae* and 27% of *A. tubingensis*. It is significant that *A. niger* accounts for nearly 70% of isolates in the US but less than 40% in Italy, highlighting significant geographic variation. Given that *A. tubingensis* does not produce FB2 mycotoxins and *A. niger* tends to produce more FB2 than *A. welwitschiae*, there appears to be a higher risk of FB2 contamination in maize attributable to black Aspergilli in the USA. Furthermore, the absence of *A. carbonarius* in both populations suggests a low risk of ochratoxin A (OTA) contamination from *Aspergillus* spp. They confirmed that FB2 production is predominant among isolates of *A. welwitschiae* and *A. niger* from maize, in accordance with previous studies on these species [46,47,48]. Although the prevalence and concentration level of FB2 synthesis in maize kernels are lower compared to fumonisin-producing *Fusarium* species, they contribute to mycotoxin accumulation. In both countries, the presence of species *A. carbonarius* was not recorded, which indicates a different population of *Aspergillus* section *Nigri* on maize compared to grapes [49].

Since *A. tubingensis*, which was the predominant species in our study, does not include FB2-producing strains, it is justifiable to expect a lower risk of FB2 contamination in maize, wheat, triticale and spelt due to black Aspergilli in Serbia. Our focus was primarily on accurately identifying *A. welwitschiae* and *A. niger* due to their known ability to produce FB2, although both species exhibit significant variability in mycotoxin production. We found five isolates of *A. welwitschiae*, which synthesized mycotoxins in very low concentrations in our preliminary testing.

Regarding the production of ochratoxin A (OTA), in Bisbal et al.’s [41] study, 51.7% of *A. tubingensis* isolates from cocoa beans were found to be producers of OTA and in higher levels than isolates belonging to *A. niger* (also from cocoa beans). Martinez-Culebras et al.’s [40] study observed a similar result—in isolates from grapes, the percentage of OTA-positive *A. tubingensis* was 53% (nine out of 17). These findings raise the question whether our *A. tubingensis* isolates have the ability to produce OTA, and if they do, how many of them and in which concentrations. Also, the question of genetic arrangements and the presence/absence of certain domains inside the genes responsible for OTA and FB2 production [50] in our strains from both species identified remains open, and we hope that in recent future it will be unraveled.

The description of *A. welwitschiae* [7] is relatively recent, limiting substantial information on its global distribution. Massi et al. [51] reevaluated the identification of 175 isolates from various sources in Brazil previously labeled as *A. niger* and discovered that half of them were actually *A. welwitschiae*. Recent reviews have emphasized the need for reevaluation of *A. niger* aggregate species in grapes, especially since many studies were published before the description of *A. welwitschiae*. Reports from different regions have shown varying predominant species contaminating grapes: *A. tubingensis* in Cyprus [52], *A. niger* and *A. welwitschiae* in South America [5,51]. On the other hand, Bian et al. [9] showed that there is a need for a reduction in species currently used in *Aspergillus* series *Nigri*, and proposed only six species out of which one should be *A. niger,* which will encompass *A. vinaceus* and *A. welwitschiae* together with *A. niger* strains. Since many studies showed that there is a certain difference between *A. welwitschiae* and *A. niger* on a molecular level and on the basis of production of different mycotoxins, we kept the name of our five identified isolates as *A. welwitschiae* until a definite change in taxonomy name is accepted worldwide.

The existence of a larger number of CaM haplotypes inside *Aspergillus* species from section *Nigri* affirms their high molecular variability [9,39,53]. In our study, we identified Serbian *A. welwitschiae* isolate from maize as a member of CaM haplotype H8, which is the most common haplotype, and in Klich et al.’s [33] study, this haplotype included 336 isolates from Brazilian and NCBI datasets, which constituted 48.3% of all tested isolates. In nine Serbian *A. tubingensis* isolates from maize and small grains (more precisely wheat, triticale and spelt), two CaM haplotypes were identified—one found in isolates from human ear and sputum as well as in dust from a mattress and in three Serbian isolates (comprising 33,33% of Serbian *A. tubingensis* isolates) and the other haplotype more frequently occurring (in our dataset, which included representatives of multilocus haplotypes from Bian et al.’s [9] study, out of 32 *A. tubingensis* isolates included, 23 bared this haplotype) in isolates such as culture type strain of *A. tubingensis* NRRL 4875, isolate from walnut kernels of *Juglans regia* from South Africa, isolate from vineyard from Yamanashi in Japan, etc., and in six Serbian isolates (comprising 66,67% of Serbian *A. tubingensis* isolates).

The pathosystem involving *Aspergillus* section *Nigri* is a significant concern in both human and animal nutrition. The mycotoxins produced by these fungi can cause a range of adverse health effects, including toxicity, immunosuppression, and cancer. The agricultural and economic impacts are substantial, as mycotoxin contamination affects both the quality and quantity of food and feed. By understanding the conditions that promote fungal growth and by implementing strategies to control contamination, it is possible to reduce the risks posed by these mycotoxins and protect human and animal health.

Given the growing global demand for safe and nutritious food, addressing mycotoxin contamination is not just an agricultural concern but a public health imperative. Enhanced monitoring, better crop management practices, and the development of resistant crop varieties could play a crucial role in mitigating the negative impact of these harmful fungal species.

## 5. Conclusions

This distribution highlights the ecological preferences of species of the genus *Aspergillus* Section *Nigri* across different agricultural products and underscores the importance of understanding their occurrence and mycotoxin production potential in food safety assessments.

In conclusion, *A. tubingensis* distributed in various agricultural products does not contribute to OTA or FB2 contamination. In contrast, *A. niger* and *A. welwitschiae*, although rarer, may be of greater concern due to their potential mycotoxin production capabilities, particularly FB2. Further research is recommended to thoroughly investigate the species distribution and profiles of mycotoxin species of the genus *Aspergillus* Section *Nigri* in agri-food products in order to ensure food safety.

## Figures and Tables

**Table 1:** Serbian isolates from maize and small grains molecularly identified in this study and their accession numbers.

Isolate	Host	Locality	Isolation Year	Accession Numbers			Identity
				ITS	CaM	RPB2	
4038	Maize	Zemun Polje	2013	OQ456471	OQ426518	PQ817115	*Aspergillus welvitschiae*
4049	Maize	Bajmok	2013	PQ810555	PQ817107	PQ817111	*Aspergillus tubingensis*
4265	Maize	Medvedja	2016	PQ810556	PQ817108	PQ817112	
4473	Maize	Zemun	2017	OQ456474	OQ426520	OQ737939	
4491	Maize	Zemun	2017	OQ456476	OQ426519	OQ737937	
4505	Wheat	Kraljevo	2018	PQ810557	PQ817109	PQ817113	
4512	Triticale	Zemun Polje	2018	OQ456477	OQ426523	PQ817116	
4797	Maize	Zemun Polje	2021	PQ810558	PQ817110	PQ817114	
4800	Maize	Zemun Polje	2021	OQ456478	OQ426521	/	
4847	Spelt	Zemun Polje	2022	OQ456479	OQ426522	OQ737936	

## Data Availability

The original contributions presented in this study are included in the article/Supplementary Materials. Further inquiries can be directed to the corresponding authors.

## Supplementary Materials

The following supporting information can be downloaded at https://www.mdpi.com/article/10.3390/foods14122146/s1, Table S1. Basic information of 45 tested isolates and their grouping on the basis of morphological traits, Table S2. ANOVA and mean squares for the virulence of all isolates, maize, wheat, triticale, and spelt isolates separately, Table S3. Mean estimates and LSD values for virulence of isolates.
