# Supplementary material for: Overview and Diversity of Fungi of the Genus Aspergillus Section Nigri on Maize and Small Grains
Source: Foods. 2025 Jun 19;14(12):2146. doi: 10.3390/foods14122146 (PMC12192094; doi:10.3390/foods14122146)
Supplement: Supplementary file 1 [file foods-14-02146-s001.zip › foods-3673215-supplementary.pdf]

Table S1. Basic information of 45 tested isolates and their grouping on the basis of morphological traits

Isolate	Plant host	Isolation year	Locality	Morpho group	Morphological characteristics of the group	Molecular identification
4037	Maize	2013	Zemun Polje	I	Vesicle size (48-78 μm); biseriate; Conidia size (2.9-3.9 μm)	*Aspergillus welwitschiae*
4038*	Maize	2013	Zemun Polje			
4205	Maize	2015	Zemun Polje			
4228	Maize	2015	Padina (Kovačica)			
4493	Maize	2017	Zemun			
4039	Maize	2013	Zemun Polje	II	Vesicle size (42-76 μm); biseriate; Conidia size (3-3.8 μm)	*Aspergillus tubingensis*
4041	Maize	2013	Bajmok			
4042	Maize	2013	Bajmok			
4049*	Maize	2013	Bajmok			
4121	Wheat	2015	Zemun Polje			
4178	Maize	2015	Zemun Polje			
4185	Maize	2015	Zemun Polje			
4204	Maize	2015	Zemun Polje			
4227	Maize	2015	Crepaja			
4265*	Maize	2015	Jabuka			
4430	Maize	2017	Zemun Polje			
4431	Maize	2017	Zemun Polje			
4473*	Maize	2017	Zemun			
4474	Maize	2017	Zemun			
4480	Maize	2017	Zemun			
4489	Maize	2017	Zemun			
4490	Maize	2017	Zemun			
4491*	Maize	2017	Zemun			
4492	Maize	2017	Zemun			
4505*	Wheat	2018	Kraljevo			
4507	Triticale	2018	Zemun Polje			
4511	Triticale	2018	Zemun Polje			
4512*	Triticale	2018	Zemun Polje			
4513	Triticale	2018	Zemun Polje			
4514	Triticale	2018	Zemun Polje			
4548	Wheat	2018	Zemun Polje			
4675	Maize	2018	Zemun Polje			
4676	Maize	2018	Zemun Polje			
4678	Maize	2018	Zemun Polje			
4701	Wheat	2020	Zemun Polje			
4735	Wheat	2021	Zemun Polje			
4797*	Maize	2021	Zemun Polje			
4800*	Maize	2021	Zemun Polje			
4807	Spelt	2022	Zemun Polje			
4810	Spelt	2022	Zemun Polje			
4816	Spelt	2022	Zemun Polje			
4821	Spelt	2022	Zemun Polje			
4847*	Spelt	2022	Zemun Polje			
4876	Maize	2022	Bačka Topola			
4877	Maize	2022	Bačka Topola			

*isolates further molecularly identified and characterized through sequencing of ITS, CaM and RPB2 genomic regions

Table S2. ANOVA and mean squares for the virulence of all isolates, maize, wheat, triticale, and spelt isolates separately

Sources of variation	d.f.	Mean Square
Isolates all	44	0.423**
Maize isolates	29	0.347**
Wheat isolates	5	0.614**
Triticale isolates	5	0,129**
Spelt isolates	5	0.059**

Table S3. Mean estimates and LSD values for virulence of isolates

Mean 4037 (maize) =	2.133	KLMN
Mean 4038 (maize) =	1.900	NO
Mean 4205 (maize) =	2.100	LMN
Mean 4228 (maize) =	2.633	DEFG
Mean 4493 (maize) =	2.800	BCDE
Mean 4039 (maize) =	2.300	IJKL
Mean 4041 (maize) =	2.733	CDEF
Mean 4042 (maize) =	2.933	BC
Mean 4049 (maize) =	2.967	BC
Mean 4178 (maize) =	2.467	GHIJ
Mean 4185 (maize) =	2.233	JKLM
Mean 4204 (maize) =	2.000	MNO
Mean 4227 (maize) =	3.033	AB
Mean 4265 (maize) =	2.833	BCD
Mean 4430 (maize) =	2.400	GHIJ
Mean 4431 (maize) =	2.567	EFGH
Mean 4473 (maize) =	2.633	DEFG
Mean 4474 (maize) =	2.833	BCD
Mean 4480 (maize) =	2.500	FGHI
Mean 4489 (maize) =	2.100	LMN
Mean 4490 (maize) =	2.467	GHIJ
Mean 4491 (maize) =	3.233	A
Mean 4492 (maize) =	2.967	BC
Mean 4675 (maize) =	2.867	BCD

Mean	4676 (maize) =	2.933	BC
Mean	4678 (maize) =	2.367	HIJK
Mean	4797 (maize) =	2.500	FGHI
Mean	4800 (maize) =	2.833	BCD
Mean	4876 (maize) =	2.367	HIJK
Mean	4877 (maize) =	2.633	DEFG
Mean	4121 (wheat) =	2.367	HIJK
Mean	4505 (wheat) =	3.000	B
Mean	4548 (wheat) =	2.733	CDEF
Mean	4701 (wheat) =	1.800	O
Mean	4735 (wheat) =	2.367	HIJK
Mean	4507 (triticale) =	2.133	KLMN
Mean	4511 (triticale) =	2.500	FGHI
Mean	4512 (triticale) =	2.000	MNO
Mean	4513 (triticale) =	2.233	JKLM
Mean	4514 (triticale) =	2.433	GHIJ
Mean	4807 (spelt) =	1.800	O
Mean	4810 (spelt) =	1.933	NO
Mean	4816 (spelt) =	2.100	LMN
Mean	4821 (spelt) =	2.133	KLMN
Mean	4847 (spelt) =	1.900	NO
